# Increased demand of urine cultures from Danish general practice: a five-year register-based study

**DOI:** 10.1080/02813432.2023.2196546

**Published:** 2023-04-13

**Authors:** Michael Adelsen Jakobsen, Mia Carøe Sørensen, Jette Brommann Kornum, Alina Zalounina Falborg, Malene Plejdrup Hansen

**Affiliations:** a Center for General Practice, Aalborg University, Aalborg, Denmark; b Department of Clinical Microbiology, Aalborg University Hospital, Aalborg, Denmark; c Research Unit of General Practice, University of Southern Denmark, Aalborg, Denmark

**Keywords:** Urine culture, general practice, urinary tract infection, microbiology testing, uropathogen

## Abstract

**Objective:**

To characterise and explore the development in the number and content of urine samples sent from general practice in the North Denmark Region to the Department of Clinical Microbiology (DCM) at Aalborg University Hospital during a five-year period.

**Design:**

A register-based study.

**Setting:**

General practice.

**Subjects:**

Urine samples received at DCM, Aalborg University Hospital from general practice between 2017 and 2022.

**Main outcome measures:**

Number and content of urine samples.

**Results:**

A total of 255,271 urine samples from general practice were received at DCM, with 76.1% being from female patients. Uropathogens were identified in 43.0% of the samples. During the five-year period, a 23.0% increase in the number of urine samples per person (incidence rate ratio (IRR) 1.23, 95% CI 1.21–1.25) was observed. A slight increase in the proportion of positive cultures (risk ratio (RR) 1.03, 95% CI 1.01–1.05) was seen. No notable change in the patient population (age, gender) was observed. Overall, *Escherichia coli* was the most identified uropathogen (60.4%) followed by *Klebsiella* spp. (8.7%) and *Enterococcus* spp. (7.7%). Distribution of the various uropathogens differed slightly depending on patient gender and age, importantly *E. coli* was less frequently observed in males aged >65 years.

**Conclusion:**

During the past five years an increasing amount of urine cultures have been requested at DCM from general practice. Importantly, the cause(s) of this increasing demand needs to be explored further in future studies.

## Key Points

Appropriate diagnostics of urinary tract infections can reduce the use of antibiotics in general practice.

From 2017 to 2022 a 23% increase per person in requested urine cultures from general practice was observed.

A slight increase in positive cultures was found, but no notable change in the patient population (age, gender) was seen.

*E. coli* was the most identified uropathogen independent of gender and age, however, the proportion differed within the various groups.

## Introduction

Antimicrobial resistance constitutes a great threat for global health and results in higher medical costs, which emphasises the importance of evidence-based diagnostics prior to antibiotic treatment [1]. Compared to other European countries, primary care in Denmark has a relatively low level of antibiotic use [2]. Even though, a notable reduction in antibiotic consumption has been reported in Denmark, primary care is still responsible for 87% of which the majority is prescribed in general practice [3].

One of the most frequent indications for antibiotic prescribing in general practice is acute urinary tract infections (UTI) [4,5]. Typical symptoms and signs of UTI, such as pollakiuria and dysuria, are weak predictors of an infection, which might lead to antibiotic overuse if treatment is based on symptoms alone [6]. Danish guidelines recommend urine examination prior to an antibiotic prescription when patients present with typical UTI symptoms [7,8]. Several diagnostic approaches are used in the management of UTIs in general practice including urine dipstick, urine microscopy as well as urine culture performed and analysed in general practice and/or sent to a local Department of Clinical Microbiology (DCM) [9]. Within Danish general practices, large variations are observed in the use of either in-house urine cultures or urine cultures requested at DCM [10].

The time until test result is available vary depending on the chosen test: within minutes (dipstick and microscopy), within 24 hours (urine culture analysed in practice) [11,12], or up to three days (urine culture sent to DCM). The delay of results on the microbiology testing increases the risk of inappropriate antibiotic prescribing including overtreatment [9]. GPs receive a fee for every test performed both in-house and sent to the local DCM [13]. A recent Danish study showed that 89% of patients presenting in general practice with symptoms and signs of acute UTI had a urine culture performed, of which 65% were performed in-house and 24% at the local DCM [9].

*Escherichia coli* is the most common uropathogen causing 75–95% of uncomplicated cases of UTIs [14]. However, other bacteria such as *Klebsiella pneumoniae, Proteus mirabilis,* and *Staphylococcus saprophyticus* are also known to cause UTI [14,15]. Despite the relatively low antibiotic prescription rate in Denmark, an increase in resistant strains of especially *E. coli* has been reported [16].

To avoid the unnecessary use of antibiotics, accurate diagnostics of UTI including rational use of diagnostic tests is of great importance [17,18]. In recent years, DCMs in Denmark have reported on the increased demand of requested urine cultures from general practice [19]. However, this increase has not yet been systematically investigated. Consequently, the aim of this study was to characterise and explore the development in the number and content of urine samples sent from general practice in the North Denmark Region to the DCM at Aalborg University Hospital during a five-year period.

## Methods

### Study design and setting

This register-based study included all urine samples sent to DCM at Aalborg University Hospital from general practice (both daytime and out-of-hours) in the North Denmark Region between 1st June 2017 and 31st May 2022.

In 2017 the population of the North Denmark Region was 587,611 inhabitants with an increase of 0.5% during the five-year study period [20].

All Danish residents have free and direct access to general practice [21]. GPs act as gatekeepers providing access to other medical specialists and hospital care. General practice is financed through taxes by a combination of capitation fees and fee-for-services [21].

### Data collection and outcomes

Urine samples were collected in general practice and delivered to DCM at Aalborg University Hospital stored in boric acid. At DCM the samples were cultured for 16-20 hours in aerobic conditions with subsequent identification of uropathogens with significant growth. In midstream urine, a positive urine culture was defined according to the European guidelines as the growth of ≥10^3^ Colony Forming Units per millilitre (CFU/mL) for the primary uropathogens (*E. coli* and *S. saprophyticus)*, ≥10^4^ CFU/mL for secondary uropathogens and ≥10^5^ CFU/mL for doubtful uropathogens [22].

Data were obtained from a database at DCM, Aalborg University Hospital containing information about all urine samples analysed at the department. The dataset included information about; the patient gender and age, date of receival of urine sample, the result of culture (positive, negative, rejected), and the type of uropathogen identified. A positive culture could contain more than one uropathogen. Cultures with more than three different uropathogens were reported as ‘polymicrobial’. Rejected urine samples included samples not analysed due to various preanalytical errors, for example use of wrong urine transport containers.

### Statistical and data analysis

Data were grouped in years. One year was defined as the period between 1st June and 31st May the upcoming year. The following age groups were used: <4 years; 4–14 years; 15–65 years; and >65 years [8,23]. Descriptive statistics were used to generate information of the number and development of urine samples and aetiology as well as patient characteristics including median age with interquartile interval (IQI). Negative binomial regression was used to estimate the incidence rate ratio (IRR) with a 95% confidence interval (CI) in modelling the number of urine samples per person with year as the independent variable. The total population in the North Denmark Region at the third quarter each year served as the offset of the model [20]. To model the proportion of positive test results and aetiology with year as an independent variable we used a generalized linear model for the binomial family estimating risk ratio (RR) with 95% CI and applying cluster robust variance at the patient level to account for possible multiple measurements per patient. Pairwise deletion was used to handle missing data when the social security number was unknown or missing. All statistical analysis were conducted using STATA/MP 17 [24].

## Results

### Population characteristics

**Figure 1.**
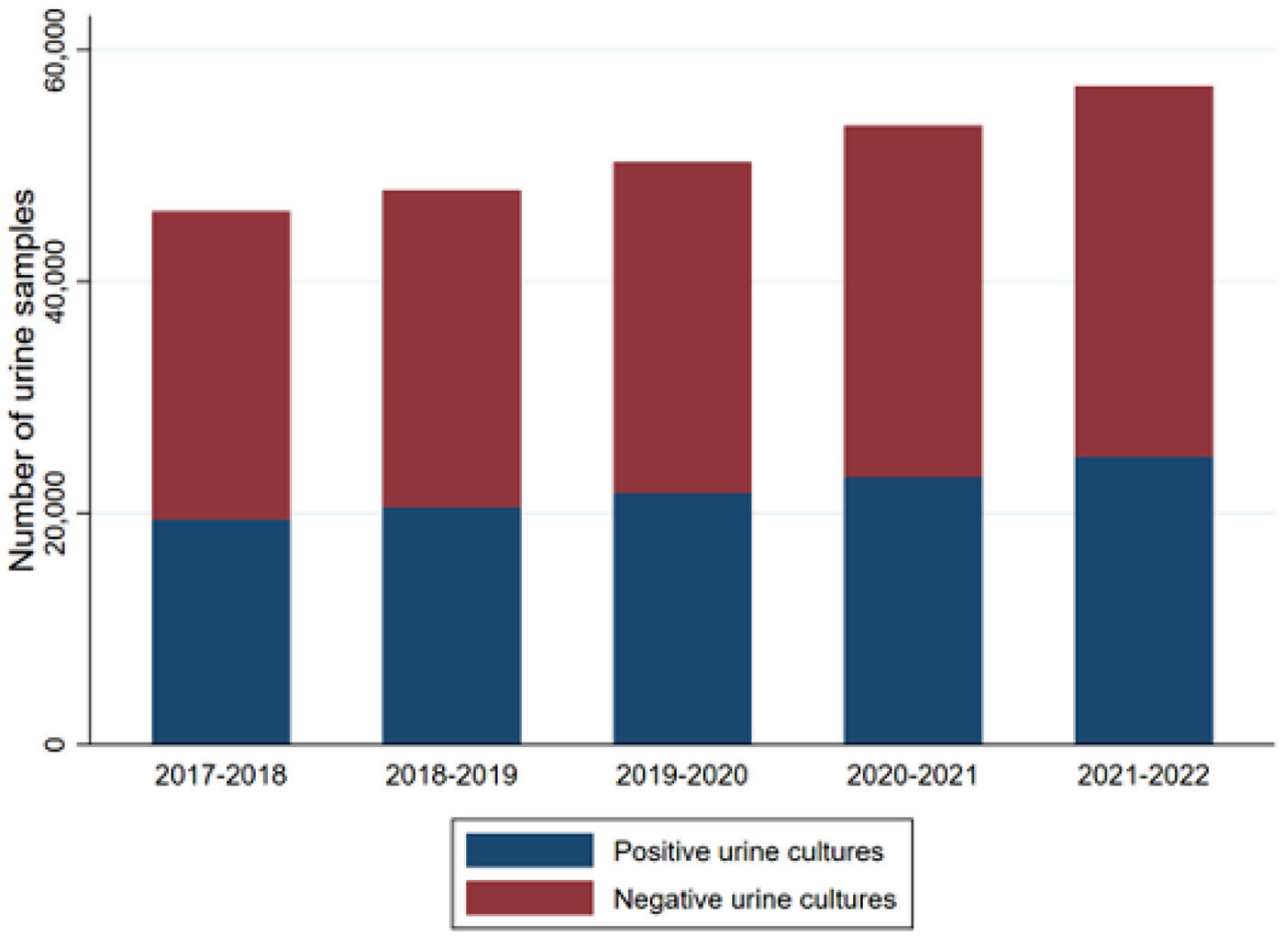
Development in the total number of urine samples including the distribution of positive and negative test results.

This study included a total of 255,271 urine samples from 108,521 patients during a five-year period (2017–2022) (Figure 1). The patient median age was 58 years (IQI 29.7-76.3) and 76.1% of samples were from females (Table 1). During the study period, 53.7% of patients had delivered one urine sample, 19.2% two urine samples, and 27.0% three or more urine samples (range 3–75).

**Table 1.** Characteristics of the study population.

	Total urine samples^a^
	(*n* = 255,271)
Gender^b^	
Females	194,214 (76.1)
Males	60,778 (23.8)
Age^b^	
<4 years	3153 (1.2)
4–14 years	10,208 (4.0)
15–65 years	132,902 (52.1)
>65 years	108,729 (42.6)

^a^Data reported as *n* (%).

^b^Urine samples without knowledge of age and gender were categorised as missing data *n* = 279 (0.1%).

Uropathogens were identified in 43.0% of the samples. The percentage of positive cultures varied significantly with age; 62.2% of cultures in the elderly (age >65 years) to 26.5% of those delivered by children aged <4 years (data not shown).

### Increase in urine samples

**Figure 2.**
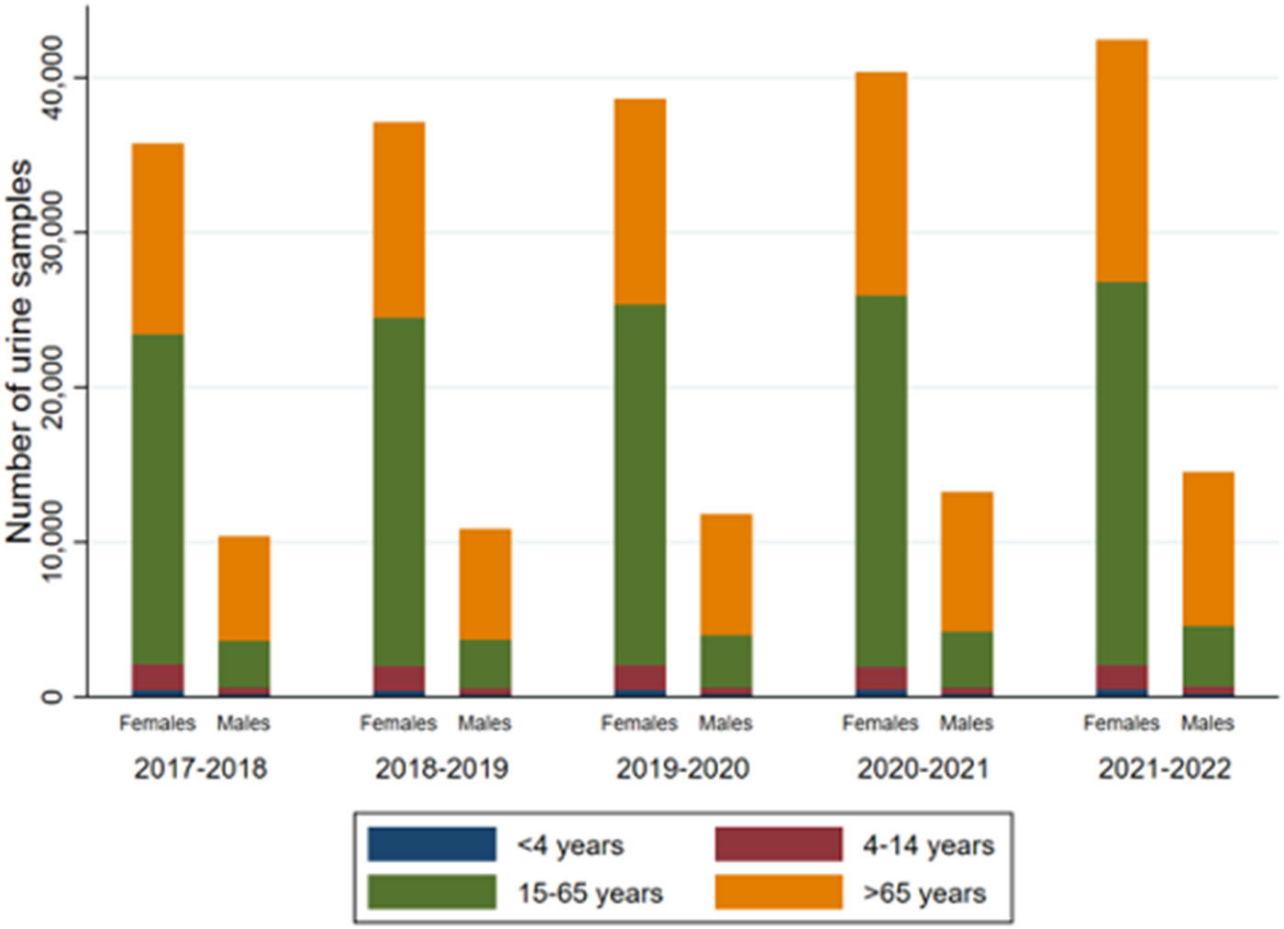
Distribution of urine samples within the age group and gender over the five-year period*. *Data regarding age and gender were missing for 279 urine samples ranging from 37 to 68 samples per year (data not shown).

Figure 1 shows an increase in the number of urine samples during the five-year period (Figure 1). The total amount of rejected urine samples per year were almost stable (range 129–165) (Data not shown). During the study period, a 23% (IRR 1.23, 95% CI 1.21–1.25) increase in the total number of urine samples per person was observed. Furthermore, a slight increase of 3% (RR 1.03, 95% CI 1.01–1.05) in the proportion of positive test results was observed. Despite an increase in the number of urine samples sent from general practice, no notable change in the patient population (age, gender) delivering urine samples was observed (Figure 2).

### Uropathogens

In total 109,749 positive urine cultures with significant growth of 117,889 uropathogens were identified during the five-year period. As shown in Table 2, the most frequently identified uropathogen was *E. coli* (60.4%) followed by *Klebsiella* spp. (8.7%)*, Enterococcus* spp. (7.7%) and *Proteus* spp. (2.8%).

**Table 2.** Uropathogens identified in urine cultures from general practice.

Bacteria species	Total^a^
*E. coli*	71,258 (60.4)
*Klebsiella* spp.	10,290 (8.7)
*Enterococcus* spp.	9121 (7.7)
Polymicrobial	5657 (4.8)
*Proteus* spp.	3294 (2.8)
Other bacteria^b^	3016 (2.6)
*Aerococcus* spp.	2677 (2.3)
*S. saprophyticus*	2539 (2.2)
*P. aeruginosa*	2446 (2.1)
Group B *Streptococci*	2340 (2.0)
*Citrobacter* spp.	2298 (2.0)
*Enterobacter* spp.	1499 (1.3)
*S. aureus*	1454 (1.2)
Total	117,889 (100)

^a^Data reported as *n* (%).

^b^Other bacteria was defined as species representing <1% in total.

**Figure 3.**
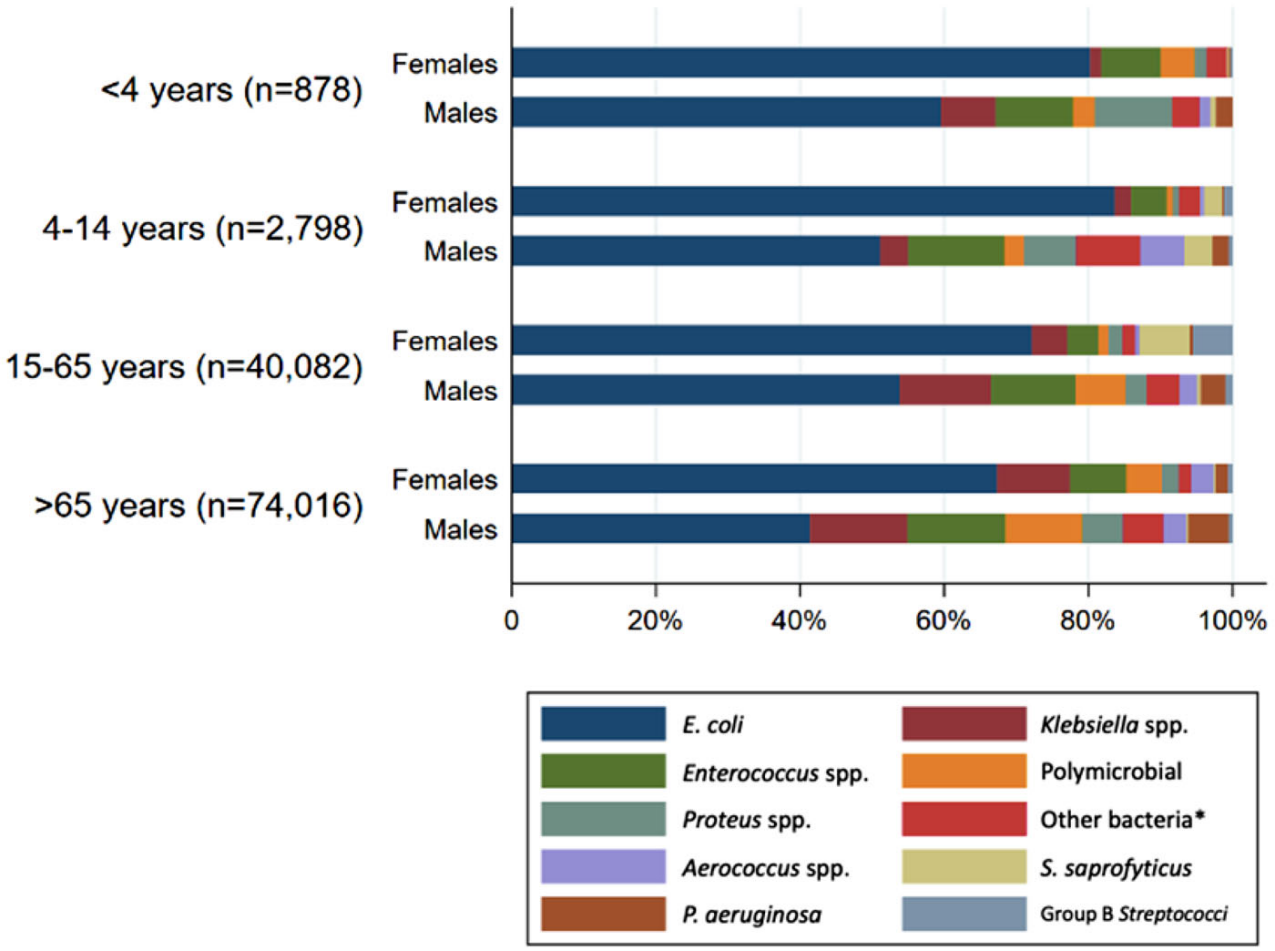
Distribution of uropathogens according to age group and gender. *Other bacteria were defined as species representing <1% in total.

No significant change in the proportion of *E. coli* (RR = 1.02, 95% CI 1.00–1.04) and remaining bacteria (RR = 1.01, 95% CI 0.96–1.07) was seen when comparing the year 2017–2018 with the year 2021–2022. However, a trend of increase in *Proteus* spp. (RR = 1.14, 95% CI 0.98–1.34) and polymicrobial (RR = 1.10, 95% CI 1.00–1.22) was noticed. Furthermore, a minor constant decrease in the proportion of *Enterococcus* spp. (RR = 0.97, 95% CI 0.96–0.99, test for trend) and *Klebsiella* spp. (RR = 0.97, 95% CI 0.95–0.99, test for trend) was observed across all five years. Distribution of the various uropathogens varied depending on patient gender and age (Figure 3). *E. coli* was most common in females within all age groups. Importantly, *E. coli* was shown to be less prevalent in patients aged >65 years, especially in males. Both *Klebsiella* spp. and *Enterococcus* spp. were more commonly identified in urine cultures from male than female patients. In addition, *S. saprophyticus* and Group B *Streptococci* were most frequently identified in females, especially in patients aged 15–65 years.

## Discussion

### Statement of principal findings

This study found a 23.0% increase in total urine samples per person sent from general practice in the North Denmark Region to DCM at Aalborg University Hospital between 2017 and 2022. A slight increase in the proportion of positive cultures was seen. During the five-year period, no notable change in the patient population delivering urine samples was observed according to age and gender. *E. coli* was the most commonly identified uropathogen followed by *Klebsiella* spp., *Enterococcus* spp., and *Proteus* spp.

### Strengths and weaknesses of the study

The result of this study is based on large sample size. All general practices, including out-of-hours, sending urine samples to urinalysis at the DCM in the North Denmark Region were included. The GPs and other healthcare workers were not aware of this investigation since data was extracted from an electronic clinical database.

In the interpretation of the results of this study, some limitations must be considered. It was assumed that indications from the DCM guideline (symptoms and signs of UTI, screening for asymptomatic bacteriuria in pregnant women, or planned instrumentation of the urinary tract) were met when general practices requested a urine culture. Furthermore, information about the various sampling methods was not available in the dataset (e.g. midstream or urethral catheterisation). Likewise, it was unknown whether guidelines regarding correct sampling technique and handling were met for each urine sample. In addition, caution must be considered in the generalisation of the results to the overall management of patients with UTIs in Danish general practice. Data provided no knowledge of symptomatology and indication leading to the performed urine culture. Also, the number of urine samples analysed in-house in general practice is unknown. Consequently, misinterpretations of the results regarding aetiology findings and study population should be kept in mind.

In the statistical analysis of the development in the rate of urine samples, the assumption of independence within urine samples was made. This approach might have caused an overestimation of the observed increase.

### Findings in relation to other studies

Consistent with previous studies, this study found that most urine samples originated from female patients [15,25,26], as well as most positive cultures were found in a patient aged >65 years [15,25,26]. This study found, as expected, *E. coli* as the most frequently identified uropathogen, independent of age and gender, supporting the Danish guidelines regarding empiric antibiotic treatment with pivmecillinam [7,8]. This finding is consistent with the results of another Danish study [16], as well as other European studies [15,26], which also found *E. coli* as the most prevalent uropathogen (range 67%–73%). In continuation, this study found that *E. coli* was less frequently identified in patients aged >65 years, especially in male patients, which compares well to the findings of Magliano et al. [26].

To our knowledge, this is the first Danish study to systematically explore the development in the number of urine cultures requested from general practice. The identified increase could possibly indicate an increase in patients with community-acquired UTIs in Denmark. A Norwegian study have reported an increase in UTI consultations in general practice between 2006 and 2015 [27]. However other explanations must also be considered, for example, in Denmark guidelines from different health authorities on the management of UTIs are not fully congruent [7,8]. The guidelines agree on recommending urinalysis prior to antibiotic treatment, however no consensus regarding type of urinalysis prior to treatment has been established [7,8]. Importantly, a recent Danish study has shown that a substantial number of patients in general practice are offered diagnostic testing for UTIs – despite no urogenital symptoms [28]. Consequently, a potential risk of overtreatment with antibiotics exists.

Between 2016 and 2018 the national program of accreditation for Danish general practices was introduced [29]. This mandatory quality improvement program did result in various organisational changes in general practices, as well as behavioural changes of both GPs and practice staff [29]. In continuation, within the past years, an increased workload has been reported in the Danish healthcare system including general practice [30]. Perhaps these changes have caused an increased demand of having urine samples cultured outside practice [9,25,31]. In addition, perhaps economic incentives have influenced the diagnostic approach for the management of patients with suspected UTIs in Danish general practice [13].

### Meaning of the study

This study found an increased demand of urine cultures at DCM from general practice, however, no obvious explanation was identified. Urinalysis at the local DCMs is associated with high-quality diagnostic as well as used for continuously national surveillance of uropathogens causing community-acquired UTIs. However, it is also known that fast and appropriate diagnostics are of great importance to secure the high-quality of patient care, targeted treatment and to avoid the unnecessary use of antibiotics [9]. More studies are needed to investigate the reasons behind this remarkably increase in request of urine cultures at DCM from general practice during the past five years.

## Geolocation information

The North Denmark Region, Denmark.

## References

[1] World Health Organization. Global action plan on antimicrobial resistance [Internet]. 2015 [cited 2022 Sep 8]. Available from: https://www.who.int/publications/i/item/9789241509763

[2] ECDC. Antimicrobial resistance in the EU/EEA: a one health response. 2022.

[3] DANMAP. DANMAP 2021 – Use of antimicrobial agents and occurrence of antimicrobial resistance in bacteria from food animals, food and humans in Denmark. 2022 [cited 2022 Nov 30]. Available from: www.danmap.org

[4] Aabenhus R, Hansen MP, Siersma V, et al. Clinical indications for antibiotic use in Danish general practice: results from a nationwide electronic prescription database. Scand J Prim Health Care. 2017;35(2):162–169. 28585886 10.1080/02813432.2017.1333321 PMC5499316

[5] Cronberg O, Tyrstrup M, Ekblom K, et al. Diagnosis-linked antibiotic prescribing in Swedish primary care – A comparison between in-hours and out-of-hours. BMC Infect Dis. 2020;20(1):616. 32819280 10.1186/s12879-020-05334-7 PMC7441551

[6] Medina-Bombardó D, Jover-Palmer A. Does clinical examination aid in the diagnosis of urinary tract infections in women? A systematic review and meta-analysis. BMC Fam Pract. 2011;12:111. 21985418 10.1186/1471-2296-12-111 PMC3207883

[7] Medicinrådet. Baggrund for Medicinrådets behandlingsvejledning vedrørende urinvejsinfektioner [Background for the Danish Medicines Agency’s treatment guidelines regarding urinary tract infections]. 2020 [cited 2023 Mar 10]. Available from: https://medicinraadet.dk/media/1xvav3sp/baggrund-for-medicinr%C3%A5dets-behandlingsvejledning-vedr-urinvejsinfektioner-vers-1-1_adlegacy.pdf

[8] Holm A, Dalmose AL, Hansen Bl, Andersen F, et al. FAQ-ta-ark om urinvejsinfektioner i almen praksis [Fact sheet for urinary tract infections in general practice] [Internet]. 2020 [cited 2022 Sep 13]. Available from: https://vejledninger.dsam.dk/fakta/uvi/?mode=visKapitel&cid=1367

[9] Córdoba G, Holm A, Sørensen TM, et al. Use of diagnostic tests and the appropriateness of the treatment decision in patients with suspected urinary tract infection in primary care in Denmark – observational study. BMC Fam Pract. 2018;19(1):65. 29769025 10.1186/s12875-018-0754-1 PMC5956889

[10] Olesen JA, Saust Lt, Bjerrum L, Lykkegaard J, et al. Audit og kursus om urinvejsinfektioner – for personale og læger i almen praksis – Region Hovedstaden [Audit and course on urinary tract infections – for staff and doctors in general practice – Capital Region]. Audit Projekt Odense. 2020. [cited 2023 Mar 15]. Available from: https://www.apo-danmark.dk/_files/ugd/43637f_e15bf2de049f4d2f8df112ed0dec3fc1.pdf

[11] Butler CC, Francis NA, Thomas-Jones E, et al. Point-of-care urine culture for managing urinary tract infection in primary care: a randomised controlled trial of clinical and cost-effectiveness. Br J Gen Pract. 2018;68(669):e268–78–e278. 29483078 10.3399/bjgp18X695285 PMC5863681

[12] Højbjerg T, Paulsen K. Digital håndbog i mikrobiologisk analyse [Digital manual of microbiological analysis] [cited 2022 Sep 19]. Available from: http://mikrobiologiskanalyse.dk/index.html?url=content.xml

[13] Praktiserende Lægers Organisation. HONORARTABEL [Table of fee]. 2022 [cited 2022 Oct 3]. p. 1–10. Available from: https://www.laeger.dk/media/owclyvfi/honorartabel_2022_oktober.pdf

[14] Gupta K, Hooton TM, Naber KG, et al. International clinical practice guidelines for the treatment of acute uncomplicated cystitis and pyelonephritis in women: a 2010 update by the infectious diseases society of America and the European society for microbiology and infectious diseases. Clin Infect Dis. 2011;52(5):e103–20–e120. 21292654 10.1093/cid/ciq257

[15] Malmartel A, Ghasarossian C. Epidemiology of urinary tract infections, bacterial species and resistances in primary care in France. Eur J Clin Microbiol Infect Dis. 2016;35(3):447–451. 26740324 10.1007/s10096-015-2560-1

[16] Córdoba G, Holm A, Hansen F, et al. Prevalence of antimicrobial resistant *Escherichia coli* from patients with suspected urinary tract infection in primary care, Denmark. BMC Infect Dis. 2017;17(1):670. 29017466 10.1186/s12879-017-2785-y PMC5635483

[17] Lindbäck H, Lindbäck J, Melhus Å. Inadequate adherence to swedish guidelines for uncomplicated lower urinary tract infections among adults in general practice. APMIS. 2017;125(9):816–821. 28585332 10.1111/apm.12718

[18] Llor C, Rabanaque G, López A, et al. The adherence of GPs to guidelines for the diagnosis and treatment of lower urinary tract infections in women is poor. Fam Pract. 2011;28(3):294–299. 21127022 10.1093/fampra/cmq107

[19] Dansk Selskab for Klinisk Mikrobiologi. Møde i UVI-arbejdsgruppen under DSKM [Meeting in UTI-working group, DSKM] [Internet]. Odense: Dansk Selskab for Klinisk Mikrobiologi; 2022 [cited 2022 Nov 30]. p. 1–3. Available from: https://dskm.dk/arbejdsgrupper/uvi/urinvejsinfektioner-moeder/

[20] Danmarks Statistik. Befolkningstal [Population] [Internet]. [cited 2022 Sep 14]. Available from: https://www.dst.dk/da/Statistik/emner/borgere/befolkning/befolkningstal?fbclid=IwAR2ZQShdoUn3gQaUp_kNcPI5jL1kFKyxCckPaguMm8131SX4IWxnoqPClR4

[21] Pedersen KM, Andersen JS, Snødergaard J. General practice and primary health care in Denmark. J Am Board Fam Med. 2012;25(Suppl 1):S34–S38. 22403249 10.3122/jabfm.2012.02.110216

[22] Aspevall O, Hallander H, Gant V, et al. European guidelines for urinalysis: a collaborative document produced by European clinical microbiologists and clinical chemists under ECLM in collaboration with ESCMID. Clin Microbiol Infect. 2001;7(4):173–178. 11422238 10.1046/j.1198-743x.2001.00237.x

[23] Holm A, Cordoba G, Sönksen UW. Urinvejsinfektioner hos ældre [urinary tract infections in elderly]. Rationel farmakoterapi nr. 10, 2016. [cited 2022 Nov 22]. Available from: https://www.sst.dk/da/udgivelser/2016/rationel-farmakoterapi-10-2016/urinvejsinfektioner-hos-aeldre?fbclid=IwAR0UCxC7DhB8gtS_oQOnfOn2iRv2D_fJd8kreHOsG_YOvy-eC08sIKAwqEI

[24] StataCorp. Stata Statistical Software: Release 17. 2021.

[25] Holm A, Siersma V, Bjerrum L, et al. Availability of point-of-care culture and microscopy in general practice – does it lead to more appropriate use of antibiotics in patients with suspected urinary tract infection? Eur J Gen Pract. 2020;26(1):175–181. 33356665 10.1080/13814788.2020.1853697 PMC7781897

[26] Magliano E, Grazioli V, Deflorio L, et al. Gender and age-dependent etiology of community-acquired urinary tract infections. ScientificWorldJournal. 2012;2012:349597. 22629135 10.1100/2012/349597 PMC3351074

[27] Haugom LEA, Ruths S, Emberland KE, et al. Consultations and antibiotic treatment for urinary tract infections in Norwegian primary care 2006–2015, a registry-based study. BMC Fam Pract. 2021;22(1):127. 34167484 10.1186/s12875-021-01470-4 PMC8229743

[28] Saust LT, Siersma V, Lykkegaard J, et al. Diagnosis and antibiotic treatment of urinary tract infections in Danish general practice: a quality assessment. Antibiotics. 2022;11(12):1759. 36551416 10.3390/antibiotics11121759 PMC9774586

[29] Kousgaard MB, Thorsen T, Due TD. Experiences of accreditation impact in general practice - A qualitative study among general practitioners and their staff. BMC Fam Pract. 2019;20(1):146. 31660860 10.1186/s12875-019-1034-4 PMC6819337

[30] PLO ANALYSE. Antallet af meddelelser i almen praksis er i voldsom vækst [Amount of messages in general practice are increasing]. 2019 [cited 2023 Mar 10]. Available from: https://www.laeger.dk/media/i4zdrraa/antallet_af_meddelelser_i_almen_praksis_er_i_voldsom_vaekst_december2019.pdf

[31] Kollerup I, Aagaard Thomsen AK, Kornum JB, et al. Use and quality of point-of-care microscopy, urine culture and susceptibility testing for urinalysis in general practice. Scand J Prim Health Care. 2022;40(1):3–10. 35023809 10.1080/02813432.2021.2022349 PMC9090341

